# The Flavonoid Quercetin Induces AP-1 Activation in FRTL-5 Thyroid Cells

**DOI:** 10.3390/antiox8050112

**Published:** 2019-04-29

**Authors:** Cesidio Giuliani

**Affiliations:** Unit of Endocrinology, Department of Medicine and Sciences of Aging, and Ce.S.I.-Me.T., University of Chieti-Pescara, 66100 Chieti, Italy; cesidio.giuliani@unich.it

**Keywords:** quercetin, thyroid, FRTL-5, AP-1, gene expressions, PGL3 vectors, pSV0-CAT vector

## Abstract

Previous studies have shown that quercetin inhibits thyroid function both in vitro and in vivo. An attempt to evaluate the effect of quercetin at the promoter level of the thyroid-specific genes led to the observation that this compound induces the basal activity of the reporter vector. Therefore, the action of quercetin has been evaluated on the basal activity of several reporter vectors: The PGL3 basic, promoter and control vectors from Promega, and a pSV-based chloramphenicol acetyltransferase (CAT) reporter vector. In the Fisher Rat Thyroid cell Line FRTL-5 thyroid cells transiently transfected, quercetin 10 μM increased the basal activity of all the reporter vectors evaluated, although the degree of the effect was significantly different among them. The analysis of the difference among the regulatory regions of these vectors identified the activator protein 1 (AP-1) binding site as one of the potential sites involved in the quercetin effect. Electromobility shift assay experiments showed that the treatment with quercetin induced the binding of a protein complex to an oligonucleotide containing the AP-1 consensus binding site. This is the first study showing an effect of quercetin on AP-1 activity in thyroid cells. Further studies are in progress to understand the role of AP-1 activation in the effects of quercetin on thyroid function.

## 1. Introduction

Quercetin (3,3′,4′,5,7-pentahydroxyflavone) is one of the most widely distributed and abundant flavonoids present in fruits and vegetables. Several studies have shown that quercetin possesses many therapeutically relevant properties, and therefore its effects have been evaluated at cellular and molecular level [1,2,3,4,5,6,7].

Several reports have shown that quercetin possesses anti-thyroid and goitrogenic effects [8,9,10,11]. Indeed, quercetin inhibits iodide organification through a thiourea-like action inhibiting thyroid peroxidase (TPO) enzyme activity and interferes with thyroid hormone metabolism, particularly through the inhibition of type I 5′-deiodinase activity [8]. Besides this, quercetin down-regulates the expression of the thyroid-specific genes sodium/iodide symporter (NIS), thyrotropin receptor (TSHR), TPO, and thyroglobulin (TG) in the FRTL-5 rat thyroid cells in continuous culture [10,11].

An attempt to evaluate the effect of quercetin at the promoter level of the aforementioned thyroid-specific genes showed a strong induction of the basal activity of the reporter gene (PGL3 Luciferase reporter basic vector) by quercetin, which interfered with the analysis of the promoter. This observation led to inquire the action of quercetin on the expression of several Luciferase reporter vectors of the PGL3 series: the basic, the promoter, and the control vectors. Furthermore, a different type of vector was also evaluated, the pSV0-CAT.

For this study, the FRTL-5 thyroid cells were used. These cells are a nontransformed rat thyroid cell line in continuous culture that represents a well-defined and reproducible in vitro model of the thyroid gland [12,13,14,15,16].

The treatment with quercetin of the FRTL-5 cells transiently transfected with the PGL3 basic, promoter and control vectors, and with the pSV0-CAT vector, increased the activity of all the reporter vectors evaluated, although the degree of the effect was significantly different among them. The analysis of the difference among the regulatory regions of the aforementioned vectors has identified the activator protein 1 (AP-1) binding site as one of the potential transcription factors involved in the quercetin effect. Indeed, quercetin treatment results in AP-1 activation in FRTL-5 cells.

## 2. Materials and Methods

### 2.1. Materials

Quercetin was from Sigma-Aldrich Co (St. Louis, MO, USA). Heat-treated, mycoplasma-free calf serum was from Life Technologies Europe (Monza, Italy). [γ-^32^P]-ATP was from Perkin Elmer Italia (Monza, Italy). Antibodies against c-fos (sc-413) and c-jun (sc-44) were from Santa Cruz Biotechnology (Santa Cruz, CA, USA). The source of all of the other materials was Sigma-Aldrich, unless otherwise specified.

### 2.2. Cell Culture

The F1 subclone of FRTL-5 rat thyroid cells (American Type Culture Collection, CRL-8305) was a gift from the Interthyr Research Foundation (Marietta, OH, USA). These FRTL-5 cells were grown in the six-hormone (6H) medium of Coon’s modified Ham’s F-12 supplemented with 5% calf serum, 2 mM glutamine, 1 mM nonessential amino acids, and the 6H mixture (6H5% medium): 1 mU/mL bovine TSH (Thyroid-stimulating hormone), 10 μg/mL insulin, 0.4 ng/mL cortisol, 5 μg/mL transferrin, 10 ng/mL glycyl-L-histidyl-L-lysine acetate, and 10 ng/mL somatostatin. These cells were diploid, between the 5th and 25th passage, and had all of the functional properties described previously [12,13,14,15,16]. Fresh 6H5% medium was added to the cells every 2 to 3 days, and they were passaged every 7 days. In individual experiments, the cells were transferred to a five-hormone (5H) medium (i.e., without TSH), again with 5% calf serum (5H5% medium).

The treatments were performed with 10 μM quercetin. In all of the experiments with quercetin, the medium was changed every 24 h, with addition of fresh medium with quercetin. Quercetin was used from a stock solution in absolute ethanol, with control cells treated with the same amount of vehicle. The final ethanol concentration was thus identical in the control and treated samples and did not exceed 0.1% (*v/v*).

### 2.3. Plasmids and Transfection

The Luciferase reporter vectors PGL3 basic, PGL3 control, and PGL3 promoter were from Promega Corp. (Madison, WI, USA). The pSV0-CAT vector was obtained from the pSV2-CAT vector by removing the SV40 (Simian virus 40) early promoter sequences [17]. The reporter vectors were transiently transfected into the FRTL-5 cells using the diethylaminoethyl (DEAE)-dextran procedure [18,19]. The cells were grown to 60% confluency, maintained in 5H5% medium for 6 days, and then returned to 6H5% medium 12 h before transfection. Twenty-four hours after transfection, the cells were treated with 10 μM quercetin or the control vehicle for 48 h.

### 2.4. Luciferase and CAT Assay

Luciferase assays were performed by using the luciferase assay system (Promega) following manufacturer instructions and a LUMAT LB 9507 Luminometer (EG & G Berthold, Bundoora, Australia). All values were normalized for total cell proteins.

Chloramphenicol acetyltransferase (CAT) assays were performed using the CAT ELISA kit (Roche Diagnostics GmbH, Mannheim, Germany) following manufacturer instructions. All values were normalized for total cell proteins.

### 2.5. Nuclear Extracts and Electrophoretic Mobility Shift Assay

FRTL-5 cells were grown to 60% confluency and maintained in 5H5% medium for 6 days to become quiescent. They were cultured again in 6H5% medium for 24 h and then treated with 10 μM quercetin or the control vehicle for 48 h. Nuclear extracts were prepared as previously described [20,21].

DNA probe was made labeling the AP-1 consensus oligonucleotide (Santa Cruz Biotechnology) with [γ-^32^P]-ATP using T4 polynucleotide kinase (New England Biolabs Inc., Ipswich, MA, USA) and purified by passing through a G-50 MicroSpin Sephadex column. The electrophoretic mobility shift assays (EMSAs) were performed as described previously [14,21]. The binding reactions were performed in high salts plus detergent and included 1.5 fmol of [^32^P]DNA, 4 μg of nuclear extracts and 1 μg poly(dI-dC) in 10 mM Tris-Cl at pH 7.9, 5mM MgCl_2_, 50 mM KCl, 1 mM DTT, 1 mM EDTA, 0.1% Triton X-100, and 12.5% glycerol in a total volume of 20 μL, with incubations at room temperature for 30 min. Where indicated, antibodies were added to the binding reactions, followed by incubation with the nuclear extracts for 20 min before the addition of the labeled DNA. Following the incubations, the reaction mixtures were electrophoresed on 5% native polyacrylamide gels at 160 V in 0.5% Tris-borate-EDTA buffer at room temperature. The gels were dried and autoradiographed.

### 2.6. Other Assays

Protein concentrations were determined using bicinchoninic acid (BCA) protein assay kits (Pierce Biotechnology Inc., Rockford, IL, USA), with crystalline bovine serum albumin (BSA) as standard.

### 2.7. Statistical Analysis

All experiments were repeated at least three times with independent batches of cells. The data are given as means ± S.D. The significance between experimental values was determined by unpaired two-tailed *t*-test, with *p* < 0.05 or better when the data from all of the experiments were considered.

## 3. Results

### 3.1. Quercetin Up-Regulates the Activity of the Following Reporter Vectors: PGL3 Basic, PGL3 Control, PGL3 Promoter, and PSV0-CAT

**Figure 1:**
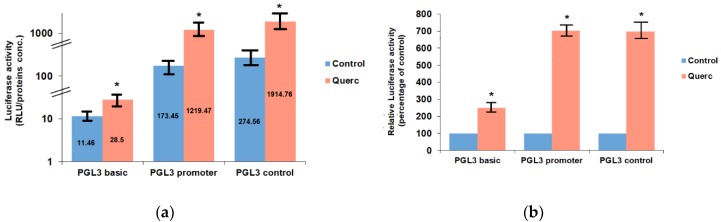
Effects of 10 μM quercetin on Luciferase activity in Fischer Rat Thyroid cell Line FRTL-5 cells transiently transfected with PGL3 basic, PGL3 promoter and PGL3 control. (**a**) Data are expressed as Relative Lights Units (RLU) to protein concentrations (proteins conc.) ratio and represent the means ± S.D. of three separate experiments; (**b**) Same data expressed relative to the control of each vector (set to 100%). Control, cells treated with the control vehicle (0.1% ethanol); Querc, cells treated with 10 μM quercetin. * *p* < 0.05 versus relevant control.

The FRTL-5 cells were grown in 6H5% medium until 60% confluent, and then switched to 5H5% medium for 6 days (i.e., without TSH) to become quiescent. They were cultured again in 6H5% medium for 12 h and then transiently transfected with the following Luciferase reporter vectors: PGL3 basic, PGL3 control, and PGL3 promoter. After 24 h from transfection, cells were treated with 10 μM quercetin for 48 h. The dose and time of the treatment were chosen based on previous studies that had shown that these treatment conditions were characterized by maximal effects of quercetin on thyroid genes expression, without toxic effects on the FRTL-5 cells [10,11]. As shown in Figure 1, quercetin treatment significantly increased the activity of the aforesaid reporter vectors. First of all, it has to be noted that there is a significant difference in the basal luciferase activities of the three vectors in the control cells (Figure 1a). This is due to the presence in the PGL3 promoter vector of the SV40 promoter region upstream of the luciferase gene (between 48 and 250 bp of the vector sequence) and to the presence in the PGL3 control vector of the SV40 enhancer region (between 2205 and 2441 bp of the vector sequence) in addition to the SV40 promoter region [22]. Therefore, the effect of quercetin was evaluated as percentage of control for each vector. This analysis revealed that quercetin treatment increased the activity of the PGL3 basic vector about 2-fold (248.69% ± 15.68% of control value), whereas it increased the activities of the PGL3 promoter and PGL3 control vectors of more than 6-fold (703.06 ± 22.3% and 697.39% ± 36.48% of control values, respectively) (Figure 1b).

These data suggested that quercetin treatment induced the activation of one or more transcription factors able to increase the activity of the PGL3 vectors. The attention was focused on the potential factors binding the SV40 promoter region, since its presence was responsible for a stronger effect of quercetin on luciferase activity (compare PGL3 promoter with PGL3 basic in Figure 1b). Nevertheless, no further significant increase was brought by the presence of the SV40 enhancer region in the PGL3 control vector compared to the PGL3 promoter vector (Figure 1b). Therefore, the putative transcription factors binding the sequence of the SV40 promoter in the PGL3 promoter vector were analyzed. This sequence, spanning from 48 to 250 bp of the vector sequence [23], was analyzed with the web tools LASAGNA-search 2.0 and AliBaba2.1 [24,25,26]. The analysis revealed several potential transcription factors binding sites. Among them, the transcription factors characterized by the best matching were AP-1, Oct-1 and Sp1, with AP-1 having the highest score, Table 1. Indeed, the SV40 promoter of the PGL vectors contains two characterized AP-1 binding sites [26], one with reverse orientation from 58 to 64 bp and the other with forward orientation from 144 to 150 bp.

The two AP-1 binding sites have an identical sequence: 5′-TGACTAA-3′, although in inverted orientation. This sequence has been first described in the human papilloma virus (HPV)-18 regulatory region, where there are also two binding sites in inverted orientation [27,28].

Potential binding sites for the transcription factors AP-1, Oct-1, and Sp1 were also identified in the backbone of the PGL3 basic vector. These data could explain the effect of quercetin in enhancing the luciferase activity even in the PGL3 basic vector.

**Figure 2:**
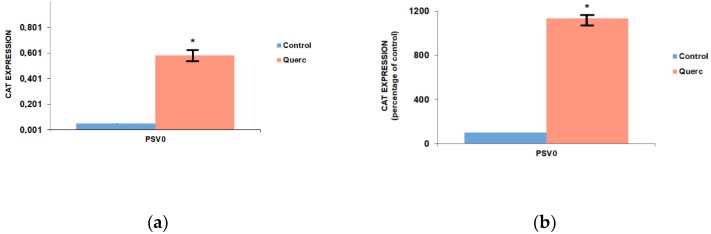
Effects of 10 μM quercetin on CAT (Chloramphenicol acetyltransferase) expression in FRTL-5 cells transiently transfected with pSV0-CAT vector. (**a**) CAT protein concentrations are normalized with respect to total cell protein concentrations and represent the means ± S.D. of three separate experiments; (**b**) Same data expressed relative to the control vector (set to 100%). Control, cells treated with the control vehicle (0.1% ethanol); Querc, cells treated with 10 μM quercetin. * *p* < 0.05 versus relevant control.

Quercetin action was also evaluated in a different reporter vector, the pSV0-CAT, a pSV2-derived vector that does not contain promoter or enhancer elements and is characterized by a very low background activity [17,29]. FRTL-5 cells were transfected with the pSV0-CAT vector, as described above for the PGL3 vectors, and treated with quercetin 10 μM for 48 h. As shown in Figure 2, quercetin treatment increased 10-fold the CAT expression compared to control activity, 1135.5% ± 24.5% of control. Surprisingly, this effect was even higher than that observed with the PGL3 control and promoter vectors.

A possible explanation of the quercetin effect on pSV0-CAT activity is the presence of cryptic promoters in the pBR322 backbone of the pSV0 vector [30,31]. Indeed, the analysis of the pSV0-CAT vector revealed the presence of the potential binding sites for the aforementioned transcription factors AP-1, Oct-1, and Sp1. Of particular interest is the presence of these binding sites in a region of about 1100 bp upstream of the CAT gene, since this region is important for the regulation of the CAT gene transcription [30,31]. Notably, a non-canonical AP-1 binding site [32,33] was present from 1025 to 1031 bp, whose sequence 5′-TGAGTAA-3′ is similar to that contained in the SV40 promoter described above (5′-TGACTAA-3′).

### 3.2. Quercetin Induces AP-1 Binding to DNA

**Figure 3:**
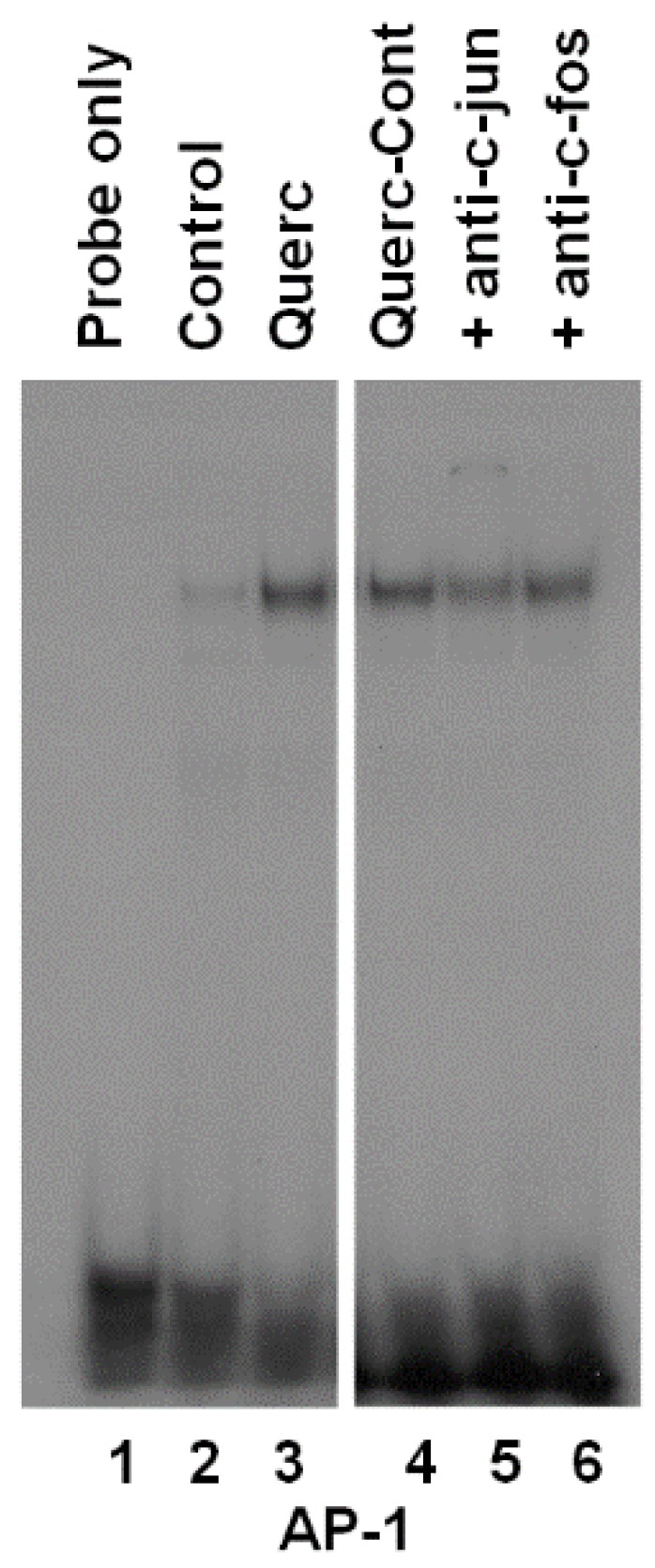
Effects of quercetin on the formation of protein/DNA complexes between FRTL-5 cell nuclear extracts and an AP-1 (Activator protein-1) consensus oligonucleotide. Radiolabeled oligonucleotide was incubated with nuclear extracts from cells cultured in 6H5% medium (control), cells treated with 10 μM quercetin (Querc) for 48 h; cells treated with quercetin plus normal rabbit polyclonal antiserum (Querc-Cont) or antibodies against c-jun (+anti-c-jun) or c-fos (+anti-c-fos). Lane 1 contains the probe alone.

The transcription factors members of the AP-1 family are involved in the regulation of thyroid growth and thyroid genes expression [34,35,36,37]. Further, quercetin has an opposite role in AP-1 activation, since it increases AP-1 activity in some in vitro models [38,39], whereas it decreases it in other models [40,41]. For these reasons, EMSA experiments were performed to evaluate the effect of quercetin on AP-1 activity in the FRTL-5 cells. Nuclear extracts from cells treated with 10 μM quercetin for 48 h were incubated with a radiolabeled AP-1 consensus oligonucleotide. As shown in Figure 3 (lane 3 versus lane 2), quercetin treatment induced the formation of a protein/DNA complex. To identify the transcription factors involved in the protein complex, nuclear extracts were preincubated with antisera to c-fos and c-jun. Preincubation of the extracts with c-jun antiserum decreased the protein/DNA complex to 52 ± 4% of control (Figure 3, lane 5 versus lane 4), whereas a slight but not significant effect was seen with the c-fos antiserum (90 ± 8,5% of control), (Figure 3, lane 6 versus lane 4). Although it was not possible to see any super shift, the decrease of the complex by the antibodies against c-jun suggests that this protein may either bind directly to the *cis*-acting element or interact with other transcription factors involved in the protein/DNA complex formation.

## 4. Discussion

The present study is the consequence of an attempt to investigate the effect of quercetin on the expression of the thyroid-specific genes at transcriptional level. Indeed, the analysis of the effect of quercetin on the NIS promoter gene inserted in the PGL3 basic vector was spoiled by a strong increase of the basal activity of the empty vector induced by the quercetin treatment. These data were surprising, as the PGL3 basic vector is believed to be transcriptionally neutral, since it lacks any viral promoter or enhancer region. However, even promoterless vectors, such as the PGL3 basic, can be transactivated by several stimuli in transiently transfected cells, due to the presence of cryptic regulatory sequences contained in the vector backbone [42,43]. Notably, quercetin had a stronger effect on the transactivation of the PGL3 promoter vector. This effect could be explained by the presence in this vector of the SV40 promoter region upstream of the luciferase gene. These data led to investigate the potential transcription factors binding sites contained in the SV40 promoter and shared by the PGL3 basic vector. Among them, AP-1, Oct-1, and Sp1 were the transcription factors characterized by the best matching. These binding sites are also present in the pSV0-CAT vector and precisely in a region particularly important for the CAT gene transcription, located about 1100 bp upstream from it [30,31]. Of particular interest is the presence in the SV40 promoter of two AP-1 binding sites, with the sequence 5′-TGACTAA-3′ in inverted orientation, which are known to play a crucial role in the control of viral transcription and replication [44]. Significantly, a similar AP-1 binding site sequence (5′-TGAGTAA-3′) is present in the CAT upstream region of the pSV0-CAT vector. It should be underlined that the AP-1 binding sites identified in the SV40 promoter and in pSV0-CAT vector with the motif 5′-TGA(C/G)TAA-3′ are identical to that observed in the regulatory region of the genital HPVs-11, -16, -18, and -59 [27,33]. This motif preferentially binds the AP-1 family subgroup jun proteins [33,45].

The present study lacks direct data confirming an AP-1 involvement in the vectors’ transactivation. However, this hypothesis is supported by the observation that quercetin can induce AP-1 binding activity on an enhancer element of the cytochrome P4501A2 gene containing the AP-1 binding site 5′-TGACTAA-3′ [46], similar to that present on the SV40 promoter and in the pSV0-CAT vector, as indicated above. However, further studies, such as mutations or deletions of the AP-1 binding sites contained in the vectors, are needed to confirm the role of AP-1 in the vector transactivation and to evaluate the role of other potential transcription factors in the quercetin effect.

The present study particularly focused on AP-1 activation for two reasons: First, the AP-1 family of transcription factors is involved in the regulation of thyroid growth and thyroid genes expression [21,34,35,36,37], second, quercetin can increase AP-1 activity in several cell types [38,39,46,47], although no data were available about thyroid cells.

This is the first study showing an effect of quercetin on AP-1 activation in thyroid cells. Although still preliminary, the experiments performed show an involvement of c-jun in the protein/DNA complex induced by quercetin. This is in agreement with previous data executed in other cell types, showing the ability of quercetin to induce the binding of members of the jun subgroup to the AP-1 binding sites [46,47].

The data reported herein are of particular relevance to understand the mechanism of action of quercetin in thyroid cells. Indeed, previous studies using the FRTL-5 cells have demonstrated that quercetin has anti-thyroid effects inhibiting the expression of the thyroid-specific genes NIS, TSHR, TPO and TG [10,11]. At least some of these effects may be mediated by AP-1 activation, as suggested by the observation that jun activation is associated with a decrease of NIS gene expression [48]. Of course, more detailed studies are needed to know which factors of the AP-1 family are activated by quercetin in thyroid cells and how they interact with the regulatory regions of the genes involved. Indeed, the AP-1 family includes several members, such as c-fos, fosB, fra-1, fra-2, c-jun, junB, junD, which can have a different effect on a single gene transcription, based on the different combinations of dimers that they can form and on the different sequences they can preferentially bind [14,21,37,49].

A final consideration that comes from the present study concerns the effect of quercetin on the basal expression of the PGL3 and the pSV0-CAT reporter vectors in the FRTL-5 cells. This observation underlines the need to always use the empty vector as control in the transfection experiments. Indeed, it is not correct to assume that a vector reported as transcriptionally neutral would not respond to cellular treatments, as also demonstrated in previous experiments performed in other cellular models [42,43].

## 5. Conclusions

This study, performed in the thyroid cell line FRTL-5, shows an interference of quercetin on the activity of the plasmid reporter vectors. These data suggest caution in the use of plasmid reporter vectors when evaluating the transcriptional effect of quercetin, due to the presence of numerous cryptic sequences for transcription factors. Moreover, the treatment with quercetin induces AP-1 activity in the FRTL-5 cells. This observation is important to understanding the mechanism of action of quercetin on thyroid cell growth and function. Further studies are in progress to evaluate the effect of quercetin on the several members of the AP-1 family and to investigate any effects on other transcription factors.

## Figures and Tables

**Table 1:** Transcription factors with the best binding match with the SV40 promoter sequence.

Transcription Factor	Score	*p* Value
AP-1	13.33	0
Oct-1	13.00	0
Sp1	12.32	0.00025

Data obtained with LASAGNA-search 2.0 [24].
